# Application of functional magnetic resonance imaging for evaluation of cartilage injury effect on knee joint function by recurrent patellar dislocation

**DOI:** 10.1097/MD.0000000000035902

**Published:** 2023-11-03

**Authors:** Yanbo Chen, Zijie Wang, Shenlu Zhang, Chengzhe Jin

**Affiliations:** a Department of Orthopedics, Nanjing First Hospital, Nanjing Medical University, Nanjing, China.

**Keywords:** cartilage injury, clinical efficacy, functional magnetic resonance imaging, recurrent patellar dislocation, vastus medialis oblique plasty

## Abstract

Explore the therapeutic effect of vastus medialis oblique plasty and the reliability and applicability of functional magnetic resonance imaging as a diagnostic method for early cartilage degeneration and injury diagnosis. From July 2020 to July 2022, there were 53 patients with recurrent patellar dislocation who met the inclusion criteria for surgery, including 34 women and 19 men, aged 11 to 53 years, with an average age of 24.4 years. After patient selection, functional magnetic resonance imaging was performed before surgery. According to the presence or absence of cartilage injury, they were divided into cartilage injury group (n = 28) and non-cartilage injury group (n = 25), and underwent vastus medialis oblique plasty. Preoperative patellar axial radiographs were performed in both groups of patients to measure the patellar tilt angle and lateral patellofemoral angle. The Lysholm, Kujala, and VAS (visual analogue scale) scores were applied to assess changes in knee joint function and anterior knee pain. All patients were postoperatively followed up. The patellar tilt angle and lateral patellofemoral angle of the 2 groups were significantly improved postoperatively (*P* < .05), with no statistical difference between the 2 groups (*P* > .05). Significant differences were observed in the VAS changes between the cartilage injury group and the non-cartilage injury group before and after operation (*P* < .05). There was a statistical difference in VAS score between the groups (*P* < .05). The changes in the Lysholm and Kujala scores before and after the operation in the cartilage injury and the non-cartilage injury groups were statistically different (*P* < .05). There was statistical difference between the 2 groups in Lysholm score and Kujala score after operation (*P* < .05). Vastus medialis oblique plasty significantly improved knee joint function and pain. Patients with cartilage injury had worse preoperative and postoperative knee function than patients without cartilage injury. Functional magnetic resonance imaging can reflect the early-stage changes in the biochemical cartilage components caused by recurrent patellar dislocation.

## 1. Introduction

Patella dislocation refers to the complete detachment of the patella from trochlear groove. In this injury, common in the young and more active population, the patellar body slips to the lateral side of the femoral lateral condyle. Patellar dislocation accounts for approximately 3% of all knee joint injuries, with an incidence of about 1‰, predominantly in the populations of 10 to 17-year-old teenagers and women.^[1]^ Recurrent patellar dislocation occurs after the first patella dislocation in some patients with internal and lateral strength imbalance of the knee joint due to damage to the medial retinaculum of the knee joint. Initial symptoms include mild mobility limitation, poor joint stability, and anterior knee pain. The later symptoms are more severe and can include habitual dislocation and patellofemoral arthritis.^[2]^

During the patella dislocation, the medial articular surface of the patella impinges on the lateral condyle of the femur, which can contribute to patellar or femoral lateral condylar cartilage fractures. Sillanpaa et al showed that, regardless of the mechanism of injury, almost all patients with patella dislocation are with joint hematoma, medial patellofemoral ligament injury, and patella medial fracture; 25% of patients suffer from osteochondral fracture.^[3]^

Currently, the diagnosis of patella dislocation in clinical practice relies mainly on imaging examinations (X-ray, computed tomography [CT], and magnetic resonance imaging [MRI]). Imaging data can provide definitive diagnosis based on the lateral dislocation of the patella, subchondral fracture, and intraarticular loose body. However, the aforementioned examinations also have shortcomings. X-rays can partly reflect the function of the knee joint, but the diagnostic sensitivity of X-rays is relatively low due to image overlap and distortion.^[4]^ Therefore, CT is often performed in clinical diagnosis. CT has a higher density resolution and is not susceptible to tissue overlap and other factors, which can clearly show patella dislocation. Compared with X-ray alone, CT can reduce the rate of missed diagnosis and misdiagnosis.^[5]^ However, CT cannot accurately diagnose soft tissue and cartilage injury. Alternatively, MRI has high soft tissue resolution with no radiation injury and multi-parameter imaging, which provides more comprehensive imaging information. Moreover, no artifacts are generated during MRI in the provision of multi-directional direct imaging.^[6]^

In recent years, with the development of high-field intensity 3.0 T MR functional imaging and the upgrading of the related software, the application of T2 mapping/Delayed gadolinium-enhanced MRI of the cartilage(dGEMRIC) was initiated for the detection of changes in the extracellular matrix of the chondrocytes and for the provision of accurate quantitative indicators, which contributes to early diagnosis of cartilage injury caused by recurrent patellar dislocation. dGEMRIC has been proposed to improve the specificity in identifying articular cartilage defects associated with knee degeneration through enhanced gadolinium binding and subsequent reduction of T1 relaxation time. dGEMRIC is a sensitive and well-validated tool for measuring cartilage changes associated with the progression of osteoarthritis (OA), even in the early stages of the disease^.[7–9]^ In this study, magnetic resonance functional imaging was performed in patients with recurrent patellar dislocation to determine whether the patellofemoral articular cartilage was damaged. In the present investigation, we also explored the effects of patellofemoral articular cartilage injury caused by recurrent patellofemoral dislocation on knee function. We aimed to establish the precise location and degree of cartilage injury by magnetic resonance functional imaging for more accurate diagnosis. Our findings extend the existing theoretical basis and provide further clinical guidance.

## 2. Materials and methods

### 2.1. General data

From July 2020 to July 2022, 53 patients with recurrent patellar dislocation who met the inclusion criteria were subjected to surgery. Of them, 34 patients were female and 19 were male, aged 11 to 53 years, with a mean age of 24.4 years.

### 2.2. Inclusion and exclusion criteria

The following inclusion criteria were applied in this study: Recurrent patellar dislocation, with a history of 2 or more lateral patellar dislocations; Patella dislocation found on clinical examination or under anesthesia.

The exclusion criteria implemented were as follows: Multiple fractures of the ipsilateral limb; open injury; History of previous operations on the ipsilateral limb; Complications with serious poor alignment (internal and valgus angles > 5°); The distance between tibial tubercle and femoral trochlear groove was greater than 20 mm; The Q angle > 20°; Complications with neurological or muscular disorders; Combinations with other ligament injuries requiring surgical reconstruction.

All patients were positive for the patellar apprehension test or “J” sign, established by preoperative physical examination. X-ray examinations showed a lateral tilt of the patella and sometimes a lateral subluxation/complete dislocation of the patella. MRI revealed subluxation or dislocation of the patella. In some cases, cartilage fractures were revealed in the X-ray and CT scans.

After patient selection, ethical approval was obtained, and patient informed consent forms were signed by all included patients. Functional magnetic resonance imaging (T2 mapping/dGEMRIC) was performed preoperatively. MR data was entered into Siemens Syngo Workplace with VVI software (Siemens Healthineers AG, Erlangen, Germany) post-processing workstation to generate T2 pseudo-color and T1 pseudo-color maps. A sagittal view through the lateral tibiofemoral joint (including patellofemoral joint) and through the medial tibiofemoral joint were selected because they would show the largest area of the cartilage. Meanwhile, a radiologist with at least 5 years of experience in MRI diagnosis of bone and joint disorders examined and analyzed these images. Based on whether cartilage injury was present, patients were divided into a cartilage injury group (n = 28) and a non-cartilage injury group (n = 25). All patients underwent arthroscopy of the knee joint + lateral release + vastus medialis oblique plasty.

### 2.3. Functional magnetic resonance imaging

A 3.0T high-field-intensity MRI scanner (Prisma, Siemens) and a 15-channel knee surface coil were used. T2 mapping was performed first, and then T1 mapping was done. After T1 mapping scanning before injection, patients were intravenously injected with 0.2 mmol/kg Gd-DTPA, and were asked to walk at a constant speed for 10 minutes. T1 mapping scan was performed the second time 60 to 90 minutes after the injection. T1 mapping scanning parameters before and after injection were the same. The positions were to be kept consistent as much as possible.

The following imaging parameters were used in our examinations: T2 mapping: the TR: 1530.0 ms, TE: 13.8/27.6/41.4/55.2/69 ms, FOV: 160 mm * 160 mm, and the matrix: 384 * 384, thickness: 4.0 mm, scanning time: 243 s, and flip angles: 180°

The T1 mapping parameters were as follows: the TR: 15.00 ms, TE: 2.7 ms, FOV: 160 mm * 160 mm, and the matrix: 384 * 384, thickness: 3.0 mm, scanning time: 276 s, and flip angles: 5°.

### 2.4. Surgical method

All patients underwent routine examination on admission. Cefathiamidine (2 g) was administered intravenously 30 minutes preoperatively to prevent infection; clindamycin (1.2 g) was used in patients with penicillin allergy. vastus medialis oblique plasty was the main surgical procedures, both of which were supplemented by release of the lateral retinaculum.

After anesthesia, the patient was placed in the supine position, and a patella displacement test was performed to confirm the lateral dislocation of the patella. Conventional disinfection and draping were performed, and air bag tourniquet was applied at 40 kPa. Anterolateral and anteromedial 5-mm incisions were made, and arthroscopic channels were established to explore the suprapatellar bursae, patellofemoral joint, intercondylar fossa, and the medial and lateral compartments. The cartilage injury of the articular surface of the patella and femur, the meniscus, and the tension of the anterior and posterior cruciate ligaments were examined. Meniscus injury and loose body were treated simultaneously during the operation. Lateral release + vastus medialis oblique plasty was performed as follows. A longitudinal 4-cm skin incision was made in the middle of the anterior patella, and the lateral release was performed first, ranging from the upper outer edge of the patella to Gerdy’s tubercle. The anterior patellar area was properly fresh with a grinding drill to facilitate the insertion of threaded rivet. Two 2.9-mm rivets with wire were driven into the patella after the hole was drilled, and the patellar insertion point of the medial femoral muscle was cut off. After transposition to the outer and lower edges of the patella, the threaded rivets were sutured and fixed. During the suturing and fixing, the fixing point was to be adjusted according to the internal and lateral tension to confirm whether the patella moved in the femoral trochlear. Finally, arthroscopic exploration was conducted to confirm the patellar movement trajectory, and no abnormalities were found. After the operation, a drainage tube was inserted into the joint cavity, the skin incision was sutured layer by layer, and the sterile dressing was covered with pressure bandage.

Postoperative management followed: knee compression bandaging was postoperatively performed in all patients, and the drainage was removed within 48 hours postoperatively. After the operation, the affected limb was fixed with an adjustable knee joint brace. One day later, ankle pump exercises and quadriceps femoris muscle isometric contraction were initiated with the affected limb. Two days later, straight leg raises with the affected limb were done to prevent quadriceps femoris muscle atrophy. The range of motion of the affected knee was limited to 0° to 45° for 4 weeks and 0° to 90° for 4 to 6 weeks. After 6 weeks, the normal range of motion of the joint was gradually restored.

### 2.5. Evaluation indicators

Functional MRI of the knee joint was completed before the operation in all patients. Axial radiographs were used to measure the patellofemoral tilt angle and the lateral patellofemoral angle. Lysholm, Kujala, and visual analogue scale (VAS) scores were applied to evaluate the knee joint function. Additionally, Lysholm, Kujala, and VAS scores were employed to assess the changes in the knee function and anterior knee pain 3 months postoperatively.

### 2.6. Statistical methods

SPSS26.0 software was used for statistical analysis. Measurement data with normal distribution were expressed as x ± s. Independent-sample *t* test was used for measurement data, and chi-square test was implemented for enumeration data. A *P* value < .05 was considered to indicate statistically significant differences.

## 3. Results

No significant difference was found in the general data (Table 1), including gender, age, and surgical side (*P* > .05).

**Table 1:** Comparison of the preoperative general data between the 2 groups.

Items	Cartilage injury group	Non-cartilage injury group	*P* value
Gender (Male/Female)	11/17	8/17	.581
Age (year)	22.5 ± 8.0	26.5 ± 12.7	.185
Left/Right	18/10	14/11	.538

**Figure 1.**
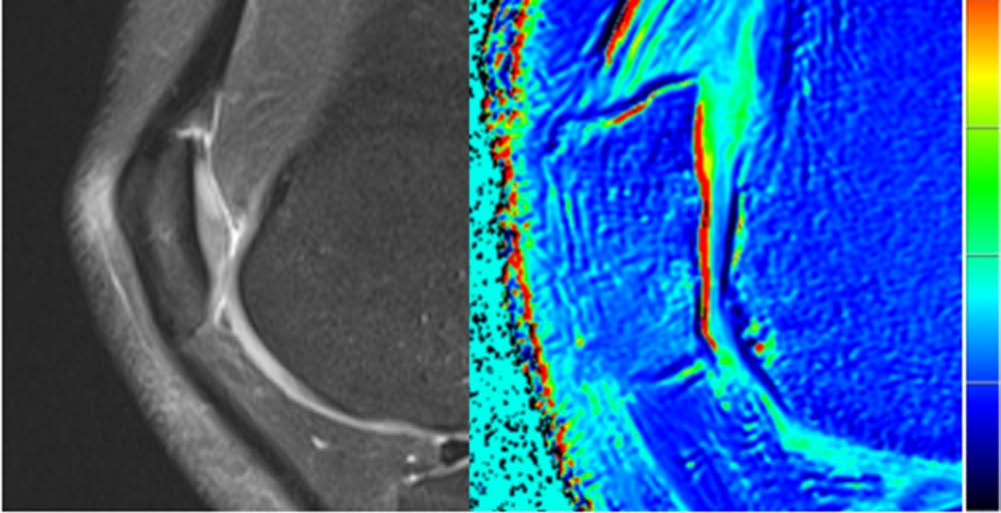
No obvious cartilage injury was found on conventional MRI and MRI functional imaging. MRI = magnetic resonance imaging.

**Figure 2.**
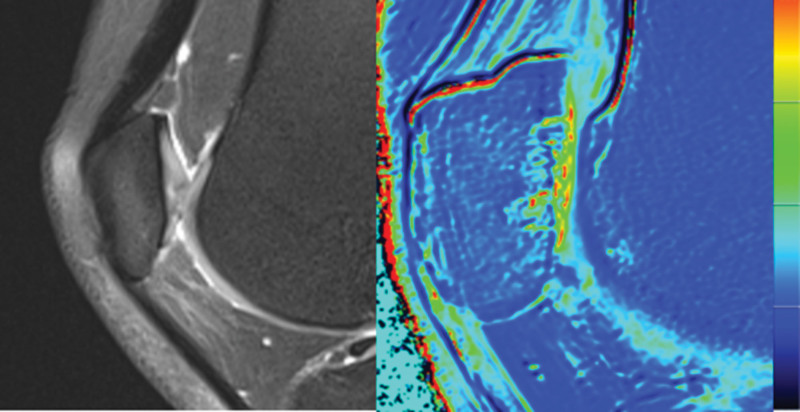
No obvious cartilage injury was detected on conventional MRI images. Functional magnetic resonance imaging showed changes in the biochemical components of the cartilage matrix and cartilage injury. MRI = magnetic resonance imaging.

Preoperative magnetic resonance functional imaging scan was performed. Patients were then divided into a cartilage injury group and a non-cartilage injury. The imaging data of the 2 groups are presented in the figures below (Figs. 1 and 2).

All patients were followed up after the operation. The surgical incisions healed (grade A) with no infection and no postoperative redislocation.

### 3.1. Comparison of the imaging parameters

There were significant differences between the preoperative and postoperative patellar tilt angle/lateral patellofemoral angle of the 2 groups (*P* < .05), but no significant differences were observed in the postoperative patellar tilt angle/lateral patellofemoral angle between the 2 groups (*P* > .05, Table 2).

**Table 2:** Comparison of the pre- and postoperative imaging parameters between the 2 groups (°, x ± s).

Group	Patellar tilt angle		Lateral patellofemoral angle	
	Preoperative	Postoperative	Preoperative	Postoperative
Cartilage injury group	17.9 ± 6.7	10.8 ± 3.1*	2.8 ± 7.3	10.3 ± 5.0*
Non-cartilage injury group	19.1 ± 5.1	11.5 ± 2.6*	2.9 ± 9.1	10.5 ± 3.2*
*P* value	.489	.334	.938	.890

* Indicates that the angle was significantly lower than the preoperative (*P* < .05).

### 3.2. Comparison of the VAS score

There were significant differences between preoperative and postoperative VAS in both of groups (*P* < .05). Patients in both groups had severe pain before operation, which was significantly relieved after operation (Table 3). There were significant differences in postoperative VAS scores different between the 2 groups (*P* < .05).

**Table 3:** Comparison of preoperative and postoperative VAS scores between the 2 groups (scores, + s).

Group	VAS scores	
	Preoperative	Postoperative
Cartilage injury group	3.8 ± 1.1	1.5 ± 0.7*
Non-cartilage injury group	2.8 ± 1.2	1.1 ± 0.7*
*P* value	.003	.027

* Indicates that the score was significantly lower than the preoperative (*P* < .05).

### 3.3. Comparison of the knee function scores

There were significant differences in Lysholm score and Kujala score before and after operation in 2 groups (*P* < .05), which was significantly improved after operation. There were significant differences in postoperative Lysholm score and Kujala score between the 2 groups (*P* < .05). The knee function score in the cartilage injury group was lower and the function recovery was slower than that in the non-cartilage injury group (Table 4).

**Table 4:** Comparison of the preoperative/postoperative knee function scores between the 2 groups.

Group	Lysholm scores		Kujala scores	
	Preoperative	Postoperative	Preoperative	Postoperative
Cartilage injury group	64.6 ± 6.0	78.2 ± 7.0*	63.7 ± 7.1	80.2 ± 4.6*
Non-cartilage injury group	69.2 ± 7.7	83.4 ± 4.7*	68.1 ± 6.3	83.7 ± 4.7*
*P* value	.020	.003	.022	.029

* Indicates that the score was significantly lower than the preoperative (*P* < .05).

## 4. Discussion

Patellar dislocation often leads to patellofemoral articular cartilage injury or even cartilage fracture. Due to the convex articular surface of the patella, during dislocation or reduction, patella collides with the femoral lateral condyle, leading to fracture of the patella or the femoral lateral condyle.^[10,11]^ In this study, magnetic resonance imaging showed that more than the half of the patients enrolled had cartilage injury after repeated dislocation of the patella, and the high incidence of cartilage injury was also closely related to the anatomy of the patellofemoral joint.^[12]^ Nomura et al^[13]^ found that the fracture site of the patellar cartilage was usually located in the central fornix of the medial side of the patella. The fracture site of the femoral lateral condyle depends on the knee flexion angle during patella dislocation. When the knee is in the extended position, the fracture often occurs on the lateral femoral trochlear ridge. Fractures may occur in the weight-bearing area of the distal lateral femoral condyle when the knee flexes more than 90°.^[14]^ In this study, patients with cartilage injury had poor knee function. Furthermore, patients with recurrent patellar dislocation had long-term repeated dislocation without intervention, especially patients with cartilage injury, which often led to patellofemoral arthritis and anterior knee pain.^[15,16]^ Duncan et al^[17]^conducted a 3-year study enrolling 414 patients and found that the cumulative incidence of patellofemoral and tibiofemoral osteoarthritis was 28.8% and 21.7%, respectively.

However, in the routine imaging examination, small cartilaginous bone mass may be missed due to poor decelopment of X-ray and CT, which will become old osteochondral fracture if not treated in time. Patients with old osteochondral fractures often have repeated knee pain, limited joint activity and joint interlocking symptoms. Moreover, the fracture area formed fibrochondral repair, the fracture fragment has become softening, partial absorption or even fragmentation, losing the best opportunity for fracture reduction and fixation. However, this study showed that cartilage injury of patellofemoral joint is very common in patients with recurrent patellar dislocation. In clinical work, X-ray and CT are commonly used as 2 convenient imaging examinations to assist diagnosis. However, the limitations of these two on diagnosis of cartilage injury cannot be ignored.

T2 mapping (T2 relaxation time mapping) is a functional, quantitative imaging technique that can reflect the content of collagen fibers in the articular cartilage, which is commonly used for evaluating articular cartilage degeneration. The relaxation time is positively correlated with the distribution of collagen fibers in the extracellular matrix of the cartilage.^[18]^ T2 mapping is highly sensitive to the changes of water and collagen fibers in the extracellular matrix caused by articular cartilage degeneration, and can effectively detect the early degenerative changes or damaged areas in the articular cartilage.^[19–21]^ In vitro studies have shown that the T2 value of the articular cartilage is positively correlated with collagen and water content presence, and increases with the severity of degeneration. In addition, some scholars combined T2 mapping with computer aided diagnosis, which can be used to detect early cartilage degeneration of OA. The impulse sequence and post-processing software of T2 mapping is easy to obtain, compatible with most MRI systems, and easily applicable in clinical practice. Cartilage injuries can be objectively and quantitatively analyzed via T2 values measurement and analysis.^[22–24]^

Due to the lower levels of anionic glycosaminoglycan in damaged cartilage than in healthy cartilage, the anionic contrast agent gadolinium can be used as a functional measure of the cartilage status.^[25]^ Delayed gadolinium-enhanced MRI of the cartilage (dGEMRIC) has been proposed to improve the specificity in identifying articular cartilage defects associated with knee degeneration through enhanced gadolinium binding and subsequent reduction of T1 relaxation time. This specificity is dependent on T1 relaxation map after injection of anionic gadolinium-based contrast agent (T1Gd) and reflects the relative concentration of glycosaminoglycan (GAG) in the cartilage matrix. After intravenous administration and subsequent diffusion into the cartilage tissue, the negatively charged side chain of proteoglycan is repelled by GAG due to the negative charge of the gadolinium-based contrast agent in the cartilage. With the progression of OA, the GAG content in cartilage decreases and more contrast agent can spread into cartilage. The presence of gadolinium reduces the T1 relaxation time so that T1Gd is proportional to the cartilage GAG content. dGEMRIC is a sensitive and well-validated tool for measuring cartilage changes associated with the progression of OA, even in the early stages of the disease.^[26,27]^

Conventional MRI spin echo sequences and proton density-weighted images show good performances only in the middle and late cartilage degeneration stages and cannot detect changes in the extracellular matrix components in the early phases of the pathological process. Therefore, in this study, we aimed to address the existing knowledge gap and correct this methodological deficiency through the implementation of functional magnetic resonance imaging. Early surgical intervention may be the best option for recurrent patellar dislocation. However, for traumatic or one-time dislocation, surgical treatment is not the only option. Functional magnetic resonance imaging can diagnose cartilage injury earlier than conventional magnetic resonance imaging.^[28,29]^ Besides, in this investigation, we also established that the knee joint function of the patients in the cartilage injury group was worse than that of the patients in the non-cartilage injury group. Patients with cartilage injury need earlier treatment, and functional magnetic resonance imaging can be used as a reliable clinical approach for the development of treatment plans.

Currently, the following surgical treatment methods are applied for recurrent patellar dislocation: Adjustment of the force line of the proximal patella: the lateral retinaculum is released; medial patellofemoral ligament reconstruction: retinaculum constriction and transposition of femoral medialis insertion; Adjustment of the distal femur force line: tendon transposition; patellar tendon operation; and transposition of the tibial tubercle. Accumulating evidence has shown that lateral ligament release or medial band compression, applied as a single surgical treatment for patellar dislocation, results in a high medium- and long-term recurrence rate. In this study, we adopted arthroscopy + lateral release + vastus medialis oblique plasty. Our postoperative results showed that this combined approach achieved a significant therapeutic effect, and the knee function of patients in the 2 groups was significantly improved postoperatively. However, in the early stage of selecting patients, careful physical examination and evaluation of imaging data are required. The treatment of patients with bone structural abnormalities, such as Q angle > 20°, TT-TG > 20 mm, and severe trochlear dysplasia of the femur often need to be combined with distal patellar rearrangement or trochlear arthroplasty to achieve better curative effect.^[30]^ Thus, such patients were not enrolled in this study for internal and lateral strength balance.

This study also has the following limitations. First, the postoperative follow-up duration was short, which provided only short-term comparative observational data of the efficacy of the applied method. In future research, long-term follow-up should be considered (1 year, 3 years, or more). Moreover, we compared the knee function scores of the 2 groups of patients were to draw more accurate conclusions. Second, the parameters and scores used to compare the efficacy of the treatment of patella dislocation were not sufficiently comprehensive.

Nevertheless, based on the results of the present investigation, magnetic resonance functional imaging can be used for early diagnosis of cartilage injury caused by patellar dislocation. Hence, patients can be treated at the early stage of the lesion, which would effectively reduce the occurrence of complications. The majority of patients with patellar dislocation are teenagers, and many patients have requirements of restoring the normal knee joint function, but they are often hesitant about operation. The normal function of the knee joint in this study was recovered by rehabilitation exercise after operation. In the long run, magnetic resonance functional imaging can be used for analyses and comparisons of the changes in the biochemical components of the knee cartilage before and after the operation to increase the effectiveness of the applied surgical method, achieving a better curative effect and providing a further theoretical basis and clinical guidance for the choice of a clinical treatment plans.In this study, after the treatment of patella dislocation with vastus medialis oblique plasty, knee function and pain were significantly improved with good clinical efficacy. Patients with cartilage injury had poorer knee function before and after the operation than patients without cartilage injury. Functional magnetic resonance imaging (T2 mapping and dGEMRIC) can reflect the changes in the early biochemical components of the cartilage caused by recurrent patellar dislocation. It is a reliable examination method that can be applied for the early diagnosis and effective clinical treatment of cartilage injury.

## Author contributions

**Data curation:** Yanbo Chen, Chengzhe Jin.

**Formal analysis:** Zijie Wang, Shenlu Zhang.

**Writing – original draft:** Yanbo Chen, Zijie Wang, Chengzhe Jin.
